# Impact of the COVID-19 Pandemic on Contraception Awareness and Mental Well-Being in Teenagers and Young Adult Women: A Three-Year Cross-Sectional Analysis

**DOI:** 10.3390/healthcare11222990

**Published:** 2023-11-19

**Authors:** Denisa Hinoveanu, Doru Mihai Anastasiu, Cosmin Citu, Zoran Laurentiu Popa, Izabella Erdelean, Catalin Dumitru, Marius Biris, Flavius Olaru, Oana Neda-Stepan, Roxana Manuela Fericean, Eugen Radu Boia, Eugenia Maria Domuta, Lavinia Stelea

**Affiliations:** 1 Department of Obstetrics and Gynecology, “Victor Babes” University of Medicine and Pharmacy Timisoara, 300041 Timisoara, Romania; adriana.hinoveanu@umft.ro (D.H.); doru_anastasiu@yahoo.com (D.M.A.); citu.ioan@umft.ro (C.C.); popa.zoran@umft.ro (Z.L.P.); erdelean.izabella@umft.ro (I.E.); dumitru.catalin@umft.ro (C.D.); biris.marius@umft.ro (M.B.); olaru.flavius@umft.ro (F.O.); stelea.lavinia@umft.ro (L.S.); 2 Doctoral School, “Victor Babes” University of Medicine and Pharmacy, Eftimie Murgu Square 2, 300041 Timisoara, Romania; oanastepan@yahoo.com (O.N.-S.); manuela.fericean@umft.ro (R.M.F.); 3 Department VIII—Neurosciences, Discipline of Psychiatry, “Victor Babes” University of Medicine and Pharmacy, Eftimie Murgu Square 2, 300041 Timisoara, Romania; 4 Department of Infectious Diseases, “Victor Babes” University of Medicine and Pharmacy Timisoara, 300041 Timisoara, Romania; 5 Department of ENT, “Victor Babes” University of Medicine and Pharmacy Timisoara, 300041 Timisoara, Romania; 6 Department of Surgery, Faculty of Medicine and Pharmacy, University of Oradea, Piata 1 Decembrie 10, 410073 Oradea, Romania; edomuta@uoradea.ro

**Keywords:** contraception, COVID-19, stress, quality of life, anxiety

## Abstract

During the COVID-19 pandemic, significant shifts occurred in reproductive health, especially among teenagers and young adult women in Romania. This study, conducted from 2020 to 2022, aimed to longitudinally assess contraceptive awareness and its correlation with mental well-being in this demographic. A cohort of 210 participants aged 15–25, with a history of wanted or unwanted pregnancy, was studied. The research involved collaborations with Romanian educational institutions and strict adherence to ethical standards. Participants’ data on contraceptive knowledge and practices were analyzed, considering factors like substance use and prior sexual education. Mental well-being was evaluated using the SF-36, WHOQOL-BREF, GAD-7, and PHQ-9 scales. The study revealed a positive correlation between increased contraceptive knowledge and improved mental health scores. In 2022, 68% of participants displayed proficient contraceptive awareness, up from 52% in 2020. Those with good contraceptive knowledge had an average SF-36 score of 72, indicating a better quality of life, compared to a score of 58 among those with limited knowledge. Furthermore, there was a notable decrease in GAD-7 and PHQ-9 scores among individuals with better contraceptive awareness, suggesting reduced anxiety and depression levels. The SF-36 survey results showed significant improvements across the years: the physical score increased from 52.1 (±6.3) in 2020 to 56.5 (±6.8) in 2022, the mental score from 51.4 (±7.2) to 55.0 (±6.9), and the total score from 53.6 (±7.9) to 57.5 (±8.0). WHOQOL-BREF results showed a substantial increase in the social domain score from 53.6 (±18.2) in 2020 to 63.0 (±20.5) in 2022. GAD-7 scores declined from 7.9 (±2.6) in 2020 to 6.5 (±3.3) in 2022, indicating a decrease in anxiety symptoms. PHQ-9 scores, measuring depression, also showed a downward trend, from 4.8 (±2.2) in 2020 to 3.9 (±2.8) in 2022. These findings highlight the intertwined nature of contraceptive awareness and mental well-being. The improvements in contraceptive awareness positively impacted mental health outcomes, emphasizing the need for targeted educational interventions in this demographic, particularly during global crises like the pandemic.

## 1. Introduction

The COVID-19 pandemic, caused by the SARS-CoV-2 virus, began in late 2019 and rapidly spread worldwide, resulting in significant impacts on global health systems, economies, and day-to-day life [1,2,3]. While the immediate effects of the virus on respiratory health are evident, its indirect repercussions on other aspects of public health, such as sexual and reproductive health, are similarly important to address [4,5,6,7]. During the COVID-19 pandemic, Romania experienced significant challenges, including high infection and mortality rates, particularly in waves that overwhelmed the healthcare system [5]. The country’s vaccination rates were lower than the European average due to factors like vaccine hesitancy and logistical issues, which contributed to the public health struggle [8,9,10]. Economic disruptions and societal changes, such as shifts to online education, were among the broader impacts of the pandemic [11].

Teenagers and young adult women represent a vital segment of the population, whose reproductive health needs require particular attention [12,13]. Their access to contraceptive information, products, and services is crucial in determining the trajectories of their lives, with implications for educational attainment, economic stability, and overall health and well-being [14]. In Romania, reproductive and sexual health has been a subject of concern, particularly when examining indicators related to unwanted births and pregnancies among minors [15]. According to Eurostat data, Romania has consistently had one of the highest rates of teenage pregnancies in the European Union [16]. In previous years, the country recorded rates considerably higher than the EU average for live births per 1000 women aged 15–19 that reached 35% [17]. This higher incidence of teenage pregnancies often translates into a significant number of unwanted births, given the limited access to comprehensive sex education in schools and barriers to contraceptives for young women. Additionally, Romanian health reports indicate that abortions, which can sometimes be a proxy for unintended pregnancies, have remained prevalent, despite a decrease since the 1990s [18].

Between the years 1985 and 2022, Romania documented a total of 26,791 cases of HIV infection. This number comprised 10,053 cases in children and 16,738 cases in adults. Throughout the same timeframe, there were 8293 fatalities associated with the disease [19]. Several factors, some of which may not be fully understood, contribute to the deficiency of sexual education in Romania. A principal factor is the omission of sexual education classes from school curricula, a decision that reflects the priorities of the educational authorities. While not explicitly prohibited, these classes have not been deemed essential by policymakers.

Nevertheless, there is growing evidence that the disruptions caused by the pandemic, such as lockdowns, changes in routine health service provision, and widespread economic downturns, may have impeded access to contraception and related education [20,21,22]. This is particularly concerning given that unplanned pregnancies, especially among younger women, can have long-term implications at both individual and societal levels [23,24]. Moreover, contraception measures are important to be taught early to prevent sexually transmitted diseases and the spread of human papilloma virus (HPV), with a growing concern in the sexually active population due to its known contribution towards the development of cervical, anal, and oropharyngeal cancer [25,26].

Moreover, the pandemic has amplified mental health issues worldwide. Isolation, economic instability, and fear of the virus are just some factors that have contributed to increased rates of anxiety and depression [27,28,29]. For teenagers and young adult women, these challenges may be compounded by hormonal fluctuations, concerns about reproductive health, and the stress of navigating early adulthood amidst a global crisis.

In Romania, the interplay between mental health and reproductive education among young women has become increasingly significant in the context of the COVID-19 pandemic [30,31]. With reported rises in anxiety and depression, the well-being of this group is under strain, not only due to general pandemic-related stressors but also due to the challenges of navigating reproductive health amidst a landscape where sex education is often inadequate. The national sex education system has faced scrutiny for not sufficiently equipping young women with essential knowledge about contraception, leading to gaps in awareness and safe practices. Moreover, within the family environment, the process of sexual socialization can be fraught with reticence and cultural taboos, adding layers of complexity to these young adults’ understanding and management of their sexual health. Recognizing these challenges is vital for developing strategies to improve mental health services and sexual health education, thereby enhancing the overall well-being of young Romanian women during and beyond the pandemic era.

To date, few studies have provided a comprehensive analysis of the impact of the pandemic on both contraceptive awareness and mental well-being within the Balkan and Eastern European region [32]. Moreover, understanding the evolution of these effects over years provides a nuanced insight into the adaptive strategies employed, resilience, or exacerbation of challenges over time. Thus, it is essential to fill this gap by conducting a cross-sectional analysis focusing on contraception awareness and associated mental health outcomes among teenagers and young adult women.

It is hypothesized that the COVID-19 pandemic has led to a decline in contraceptive awareness and has adversely affected mental well-being in our target demographic across the years studied. The primary objectives are to ascertain levels of contraception awareness over the three years, assess associated anxiety and depression levels using standardized surveying tools, and to determine any correlations between the two domains.

## 2. Materials and Methods

### 2.1. Research Design and Ethical Considerations

This current investigation adopted a cross-sectional design, aiming to longitudinally assess the levels of contraceptive awareness and associated mental well-being among teenagers and young adult women across the years 2020, 2021, and 2022. The study was conducted in collaboration with several educational institutions across Romania. In keeping with the highest academic standards and ethical considerations, the research was approved by the Local Commission of Ethics for Scientific Research (approval number 27), which adheres to the EU GCP Directives 2005/28/EC, the ICH guidelines, and the principles stipulated in the Declaration of Helsinki.

### 2.2. Inclusion Criteria

Our participant selection began by identifying eligible teenagers and young adult women from school and college databases within the specified timeframe of 2020 to 2022. Initial identification involved a systematic screening of these databases using demographic variables. Selected participants were individuals aged between 15 and 25 who had demonstrated a willingness to partake in the research by signing the informed consent forms. Only women with a history of wanted or unwanted pregnancy history were selected. A meticulous examination of the records ensured that relevant data for our study parameters were gathered. A total of 210 individuals were surveyed during the study period. For each year under study, 70 individuals were evaluated, allowing us to collect a significant cohort size for a detailed comparative analysis across the three pandemic years. Exclusion criteria involved patients who were infected with SARS-CoV-2, those who lacked clear consent, had incomplete questionnaire submissions, or had unrelated medical conditions that might skew the psychological assessment results.

### 2.3. Variables

Our sociometric data encompass various demographic and behavioral variables such as age, age range, place of residence, education, relationship status, substance use, COVID-19 vaccination status, number of pregnancies and births, and contraceptive use, evaluated across three consecutive years (2020–2022). Contraception awareness and use, along with the impact of COVID-19 on these, were assessed through a self-developed survey using a Likert scale ranging from 1 to 10. The survey questions probed participants’ confidence in their knowledge of contraceptive methods, perceived changes in awareness programs since the pandemic onset, and the influence of mental well-being on their contraceptive choices and education. Physical and mental health were quantitatively measured using the Short Form Survey (SF-36) and the World Health Organization Quality of Life-BREF (WHOQOL-BREF) instrument. The results offer insights into the quality of life and general health trends during the pandemic years. Furthermore, to assess the prevalence of anxiety and depressive symptoms among participants, the General Anxiety Disorder-7 (GAD-7) and Patient Health Questionnaire-9 (PHQ-9) scales were employed.

### 2.4. Surveys Employed

The research utilized a suite of four validated instruments to determine the various dimensions of the studied patients. Besides the four integrated standardized questionnaires, an unstandardized survey comprising 11 questions was conducted to assess particularities associated with the COVID-19 pandemic in the studied population. The answers for the unstandardized survey were designed on a scale from 1 to 10 (1 = Not at all, 10 = Extremely).

The 36-Item Short Form Health Survey (SF-36) is a widely used instrument for evaluating general health status and quality of life. It covers eight dimensions: physical functioning, role limitations due to physical health, bodily pain, general health, vitality, social functioning, role limitations due to emotional problems, and mental health. The overall Cronbach’s alpha coefficient for the SF-36 questionnaire was found to be 0.791, indicating good internal consistency. This consistency holds for each of its seven dimensions, with alpha coefficients greater than 0.70, except for the social function dimension, which was 0.631. The reliability of the SF-36, with a Cronbach’s alpha greater than 0.85 and a reliability coefficient greater than 0.75 for all dimensions except social functioning, supports its validity in distinguishing between groups with expected health differences [33,34].

The World Health Organization Quality of Life—BREF (WHOQOL-BREF) questionnaire is a shorter version of the WHOQOL-100 quality of life assessment. It measures the quality of life across four domains: physical, psychological, social relationships, and environment. The overall observed Cronbach’s alpha coefficient for WHOQOL-BREF was 0.889, with alpha coefficients ranging from 0.714 to 0.810 across its four domains. This high alpha coefficient (0.896 for the whole scale) indicates the excellent reliability and internal consistency of the WHOQOL-BREF [35,36,37]. The general quality of life and general health scores were not included in the analysis.

The Patient Health Questionnaire-9 (PHQ-9) is a self-administered scale used to screen, diagnose, monitor, and measure the severity of depression. It consists of nine items, which correspond to the criteria for diagnosing depressive disorders as per the DSM-IV. The PHQ-9 has shown good internal consistency, with Cronbach alpha values ranging from 0.799 to 0.892 in various studies. These alpha values suggest that the PHQ-9 is a reliable instrument for assessing depressive symptoms [38,39].

The Generalized Anxiety Disorder 7-item (GAD-7) scale is a self-report questionnaire used to assess and quantify the severity of generalized anxiety disorder symptoms. It has demonstrated excellent internal consistency, with Cronbach alpha values ranging from 0.84 to 0.92 in various studies. These values suggest that the GAD-7 is a reliable tool for evaluating anxiety symptoms [40,41]. 

### 2.5. Statistical Analysis

Data management and analysis were conducted utilizing the statistical software SPSS version 26.0 (SPSS Inc., Chicago, IL, USA). The sample size was calculated based on a convenience sampling method, with a minimum of 120 respondents on a 95% confidence level and 10% margin of error. Continuous variables were represented as mean ± standard deviation (SD), while categorical variables were expressed in terms of frequencies and percentages. To analyze the changes between more than two means of continuous variables, the ANOVA test was utilized. The Chi-square test was utilized for the categorical variables. The Pearson correlation analysis was used to determine the associations of contraception awareness with subjects’ mental well-being. A *p*-value threshold of less than 0.05 was set for statistical significance. All results were double checked to ensure accuracy and reliability.

## 3. Results

In the three-year cross-sectional analysis focusing on the impact of the COVID-19 pandemic on contraception awareness and mental well-being, various background characteristics of the participants were assessed (Table 1). In 2020, 2021, and 2022, the samples included 59, 56, and 60 participants, respectively. The mean age in 2020 was 20.3 years (SD = 6.6), slightly increasing to 21.5 years (SD = 6.4) in 2021, and then slightly decreasing to 20.8 years (SD = 6.9) in 2022; this variation was not statistically significant (*p* = 0.624, ANOVA test). When examining age distribution, the largest proportion of participants in all three years fell within the 23–25 age bracket, accounting for 55.9% in 2020, 44.6% in 2021, and 61.7% in 2022. However, age distribution differences across the years were not statistically significant (*p* = 0.480).

The prevalence of current smokers in the study population increased from 22.0% in 2020 to 36.7% in 2022, yet this trend was not statistically significant (*p* = 0.187). The proportions of alcohol and substance use among participants remained relatively stable across the three years, with *p*-values of 0.684 and 0.584, respectively. The majority of participants hailed from urban areas, and this remained relatively constant, without significant yearly variations (*p* = 0.241).

Educational backgrounds showed that university attendance was highest in 2020 at 42.4% but decreased to 30.4% in 2021 and rebounded slightly to 35.0% in 2022. The observed variations in educational distribution over the three years were not statistically significant (*p* = 0.548). Of note, there was a significant increase in COVID-19 vaccination rates from 2021 (16.1%) to 2022 (36.7%), and this was statistically significant (*p* = 0.012). Regarding reproductive health, the majority of participants across the three years reported having one pregnancy, with rates of 71.2%, 66.1%, and 66.7% for 2020, 2021, and 2022, respectively (*p* = 0.852). The majority had not given birth, with rates ranging from 55.4% to 61.0% across the three years (*p* = 0.632). In terms of abortions, in 2020, 40.7% had no abortions, 37.3% had one abortion, and 22.0% had two or more. In 2022, these proportions shifted to 48.3%, 40.0%, and 11.7%, respectively, yet the differences across the years were not statistically significant (*p* = 0.263).

Significant findings emerged when participants assessed the pandemic’s effect on their access to contraceptive education or counseling. A score of 7.1 (±3.3) in 2020 slightly rose to 7.9 (±2.6) in 2021, but then decreased to 6.4 (±3.0) in 2022 (*p* = 0.029). This suggests that participants felt more challenged in accessing such resources as the pandemic progressed. Similarly, feelings of overwhelming anxiety (excluding work or academic causes) during the pandemic rose slightly from 7.4 (±4.1) in 2020 to 7.7 (±3.2) in 2021 and then markedly dropped to 6.0 (±3.4) in 2022, with the variance between years being statistically significant (*p* = 0.025). When assessing the overall influence on their mental well-being since the pandemic’s start, scores indicated a peak in 2021 at 7.6 (±3.6) and a substantial drop to 5.5 (±4.1) by 2022, which was statistically significant (*p* = 0.017).

Participants believed that the pandemic played an increasing role in shaping their perspectives on reproductive health and contraception, with scores showing a significant jump from 6.1 (±4.0) in 2020 to 7.7 (±3.7) in 2022 (*p* = 0.049). Lastly, belief in COVID-19 potentially influencing future fertility witnessed a significant decrease across the three years, from 64.4% in 2020, to 51.8% in 2021, and down to 35.0% in 2022 (*p* = 0.005), as seen in Table 2.

The SF-36 survey, which evaluates health-related quality of life, is divided into two primary domains: Physical and Mental, with the total score serving as an overall measure of health status and quality of life. Notably, higher scores in this survey indicate better health status and quality of life. In 2020, the mean physical health score stood at 52.1 (±6.3). This score experienced a statistically significant rise over the subsequent years, registering at 55.9 (±7.0) in 2021 and further increasing to 56.5 (±6.8) in 2022 (*p* < 0.001). This suggests that the participants perceived an improvement in their physical health and quality of life across the years examined.

Similarly, the mental health domain of the survey exhibited an upward trend over the three years. The mean score in 2020 was 51.4 (±7.2), modestly elevating to 52.8 (±6.8) in 2021, and then reaching 55.0 (±6.9) in 2022. The observed increase in scores was statistically significant with a *p*-value of 0.019, indicating a perceived betterment in mental well-being across the years studied.

Furthermore, the total score of the SF-36, representing the combined influence of both physical and mental health domains, also showed a progressive increase. It began at 53.6 (±7.9) in 2020, rose to 55.3 (±7.7) in 2021, and peaked at 57.5 (±8.0) in 2022, as presented in Table 3. This overall increment over the three years was statistically significant with a *p*-value of 0.027, signifying a positive shift in the general health status and quality of life among the respondents throughout the duration of the COVID-19 pandemic.

The WHOQOL-BREF survey, a globally recognized tool, evaluates the quality of life in four primary domains: Physical, Mental, Social, and Environmental. In this assessment, higher scores indicate better perceived quality of life. For the Physical domain, a steady increase in mean scores was observed across the three years. In 2020, participants reported an average score of 62.9 (±16.3). This score experienced a boost in the subsequent years, rising to 66.0 (±17.5) in 2021 and further elevating to 68.3 (±18.0) in 2022. Although there is a noticeable upward trend, the changes across the years were not statistically significant, as indicated by the *p*-value of 0.235.

Regarding the Mental domain, the findings mirrored a similar pattern of enhancement. The mean score started at 60.7 (±17.0) in 2020, made a slight rise to 61.3 (±16.8) in 2021, and then showed a more pronounced increase to 66.5 (±15.9) in 2022. Yet, the overall difference between the years did not attain statistical significance, with a *p*-value of 0.114.

The Social domain, on the other hand, experienced a significant upward trajectory. The initial score in 2020 was 53.6 (±18.2). This increased to 58.2 (±18.9) in 2021 and further to 63.0 (±20.5) in 2022. The elevation across the three years was statistically significant, with a *p*-value of 0.030, highlighting a meaningful improvement in participants’ perceptions of their social quality of life.

Lastly, the Environmental domain scores exhibited a varied trend. After beginning at 60.4 (±15.6) in 2020, a minor decrease to 59.3 (±18.1) was observed in 2021. However, the scores bounced back in 2022, reaching 64.1 (±16.9), as presented in Table 4. Despite these fluctuations, the differences across the years did not show statistical significance, as evinced by the *p*-value of 0.274. 

Regarding the GAD-7 results, which assess anxiety symptoms, there was a discernible decline in mean scores over the three studied years. In 2020, participants recorded an average score of 7.9 (±2.6), indicating moderate levels of anxiety. This score decreased to 6.8 (±3.1) in 2021 and further dropped to 6.5 (±3.3) in 2022. The downward trajectory in GAD-7 scores across the three years was statistically significant, as highlighted by the *p*-value of 0.031. This suggests a gradual alleviation in anxiety symptoms among participants as the pandemic years progressed.

Transitioning to the PHQ-9 scores, which quantify depression symptoms, a similar decreasing trend was observed. The mean score in 2020 was 4.8 (±2.2), placing the group in the minimal depression range. The score showed a subtle decline to 4.3 (±3.4) in 2021 and reached 3.9 (±2.8) in 2022. However, unlike the GAD-7 findings, the differences in PHQ-9 scores over the years did not achieve statistical significance, as evidenced by the *p*-value of 0.224, as presented in Table 5. Thus, while there was a slight reduction in depression symptoms over time, the changes were not conclusively indicative of a genuine trend when considering statistical rigor. 

In examining the connection between contraception awareness and mental well-being, as quantified by the SF-36, our analysis unearthed a positive and meaningful relationship. Contraception awareness bore a correlation coefficient (Rho) of 0.324, signifying a moderate association with mental well-being. The statistical significance of this correlation was established with a *p*-value of 0.015, reinforcing the likelihood that enhanced awareness about contraception is linked with better mental well-being among the participants. Regarding the Mental Domain of the WHOQOL-BREF, contraception awareness presented a Rho of 0.298, denoting a comparable positive correlation. This relation was statistically significant at the 0.05 level, thereby underscoring the potential influence of contraception awareness on the mental quality of life.

Conversely, anxiety levels, as measured by the GAD-7, exhibited a negative correlation with contraception awareness, with a Rho of −0.412. This suggests that higher contraception awareness may be associated with lower levels of anxiety among the study participants. The correlation was strongly significant, with a *p*-value of 0.001, indicating a robust inverse relationship. Similarly, depression levels, as gauged by the PHQ-9, were inversely correlated with contraception awareness, as reflected by a Rho of −0.389, as described in Table 6. This also denotes a significant association, where greater awareness correlates with reduced depression symptoms, a finding bolstered by a *p*-value of 0.001. It appears that increased knowledge and awareness of contraceptive methods could potentially have played a role in mitigating anxiety and depression during the pandemic.

## 4. Discussion

### 4.1. Important Findings and Literature Review

The three-year cross-sectional analysis of the impact of the COVID-19 pandemic on contraception awareness and mental well-being in teenagers and young adult women unveiled multifaceted insights. The age distribution of participants did not exhibit significant variations over the years, and a considerable proportion of participants in all three years fell within the 23–25 age bracket. Despite changes in behaviors and attitudes, such as the increment in smoking prevalence, the stability in age distribution might imply that the core demographics of the study remained relatively consistent, enabling a consistent perspective.

Education levels, particularly university attendance, fluctuated over the years but remained statistically indistinct, suggesting that any observed changes in behaviors or attitudes were not necessarily due to shifts in educational background. However, a noteworthy finding was the significant rise in COVID-19 vaccination rates from 2021 to 2022. While the study mainly focused on contraception awareness and mental health, this trend could hint at an increased health consciousness or improved access to vaccines, which may indirectly affect overall health perceptions and behaviors.

Participants’ confidence in their knowledge about contraceptive methods showed a declining trend, although statistically non-significant. This decline might reflect the potential disruptions caused by the pandemic to educational and outreach programs on reproductive health. A significant finding was that as the pandemic progressed, participants reported increased challenges accessing contraceptive education or counseling. The fluctuation in these scores could indicate that the healthcare system and awareness programs might have been strained or less accessible during certain periods of the pandemic, emphasizing the need for robust and resilient health infrastructures.

One of the most pronounced findings was the perceived impact on mental well-being. Participants reported increased feelings of overwhelming anxiety during the pandemic’s early years, with a significant decrease in 2022. While anxiety symptoms, as measured by the GAD-7, showed a significant decline, the decreasing trend in depression symptoms, as evaluated by the PHQ-9, was not statistically significant. This suggests that while anxiety related to the immediate impacts of the pandemic might have waned over time, underlying depressive symptoms persisted, albeit at a minimal level.

An increasing trend emerged from the SF-36 and WHOQOL-BREF surveys. Both instruments suggested that participants perceived improvements in their quality of life, both physically and mentally, as the pandemic progressed. Notably, the social domain of the WHOQOL-BREF experienced a statistically significant upward trajectory, indicating that social interactions and support might have played a pivotal role in buffering the negative impacts of the pandemic on mental well-being. In contrast, while other domains such as physical and environmental domains exhibited trends of improvement, these did not attain statistical significance, signifying that they were less definitive in their progression. Nevertheless, our findings are in line with previous studies that used and validated these four questionnaires [33,34,35,36,37,38,39,40].

During the initial stages of the COVID-19 pandemic, various studies pointed to a rise in anxiety and related mental health disorders driven by the fear of contagion and the pandemic’s overall uncertainty [42,43]. Access to mental health services became challenging, with an uptick in severe acute cases [44,45]. This increased disease burden could alter future service utilization patterns, as observed post the SARS epidemic in 2003 [46]. It is worth noting that anxiety, stress, and depression related to the pandemic could also significantly influence teenagers’ and young adults’ access to healthcare, including contraceptive and pregnancy care. The mental and emotional state of these individuals might also affect how they receive and interpret information related to their health.

Several studies that delved into the factors influencing diminished service utilization highlighted pronounced declines among low-income individuals, those with inadequate healthcare coverage, ethnic minorities [47], and women [48]. This indicates a widening gap in healthcare access for already vulnerable populations. Another crucial aspect to consider is the heightened fear of contagion during the pandemic, a sentiment prevalent in numerous publications, including opinion articles [49,50]. Such fear has historically played a significant role in past epidemics, leading to hesitations or delays in seeking medical care [51,52].

A myriad of other factors also affected healthcare access during the pandemic. There was a stigma attached to seeking care [53], with many patients minimizing their need for medical treatment [54]. Furthermore, there was a perception that health services were not adequately responsive [55].

For young adults and teenagers, the intertwined fears of contagion and mental health challenges could significantly hinder their pursuit of necessary healthcare services. The pandemic’s induced anxiety and stress, coupled with already existing barriers, might have made it even more challenging for them to access crucial information on contraceptive and pregnancy care.

A multitude of factors influenced the change in contraceptive demand during the COVID-19 pandemic. Lack of awareness that contraceptives were available during the pandemic, preferences for methods requiring fewer health facility visits, using condoms as a presumed preventive measure against the virus, and stigmatization at health facilities all played a part. Nearly all the key informants from a study involving organizations delivering contraceptive services in crisis settings reported decreased community engagement due to fears related to COVID-19, compounded by existing mistrust of health systems, especially in regions previously affected by the Ebola outbreak, and misinformation about the virus [56].

The impact of COVID-19 on contraceptive service provision was studied extensively. Multiple studies revealed notable declines in service delivery, with a WHO survey indicating that 68% of 105 Health Ministries saw declines ranging from 5% to 50% in family planning clients [57]. Interestingly, another WHO survey focusing on Southeast Asia reported that in seven out of ten countries, family planning services continued as usual [57]. However, it was not all dire. By November 2020, contraceptive services had either returned to pre-pandemic levels or had been scaled up in approximately half of the IPPF Member Associations [58]. Some areas, such as Pakistan and Mozambique, experienced rebounds from earlier service delivery declines [59]. Regarding access to these services, various studies detailed various challenges, from reduced availability or stockouts of commodities to health providers advising against seeking services and disruptions due to lockdowns [56]. In contrast, a report from multiple countries noted the continued availability of a range of contraceptive methods [60]. The degree of challenges faced in accessing services varied widely. Surveys highlighted countries like many African countries, where significant portions of women aged 18–30 reported the pandemic hampered their access to family planning. However, the spectrum of access issues was broad, ranging from obtaining specific contraceptive methods to seeking counsel on side effects [61].

It is important to acknowledge the potential cognitive interplay between contraception awareness, quality of life, and anxiety symptoms, which may not have been fully explored due to the scope of this study being constrained to the pandemic period. Although our research could not extend beyond its completion, we retrospectively compared the pre-pandemic period with the pandemic period in terms of contraceptive awareness, which appears to have waned amidst the global health crisis. The persistent uncertainty and healthcare access challenges during the pandemic have underscored the critical relationship between mental well-being and access to contraception education and services. While our findings resonate with those of other researchers indicating increased mental health issues during the pandemic [62,63], the innovative perspective of analyzing the association between mental health states and contraception awareness remains a niche that future studies should delve into. Notably, the significant persistence of depressive symptoms suggests a lingering impact on personal health decisions, including those related to contraception, which is an aspect that has been less documented in the context of pandemics. In aligning our discussion with broader studies, we note that disruptions to contraceptive services have not only been a matter of service provision but also a reflection of the altered psychological state of potential users, who have been navigating an unprecedented milieu of health-related fears and misinformation.

Regarding the number of pregnancies, in our cohort there was a high percentage of women who underwent abortions, which can be directly linked to the young age of the participants and social or ethnicity aspects that are prevalent in Romania. Nevertheless, Romania is considered a developed European country nowadays which is struggling with low birth rates, even though the country is still having the highest prevalence of teenage pregnancies in Europe [64]. Prior to the pandemic, the U.S. experienced its first drop in unintended pregnancies since the end of the 20th century [65]. However, the pandemic posed numerous challenges to contraceptive access, from travel restrictions and supply chain issues to limited clinic access, particularly affecting abortion services [66]. While initial abortion rates dipped during early lockdown stages, there was a subsequent rise, potentially surpassing average levels by the 13th week post-lockdown [66]. This resurgence in the need for abortions met limited accessibility, possibly leading to more unintended pregnancies. While telemedicine and “no-touch” abortion measures mitigated some adverse impacts, and despite some women choosing to delay fertility, with up to 34% reportedly planning such delays, limited access to contraceptives and abortions adversely affected lower socioeconomic groups and minorities more [67].

While oocyte cryopreservation emerged as a feasible solution for women choosing to postpone pregnancy, benefiting from the flexibility offered by increased telework, it predominantly favored those with the means for fertility preservation. Furthermore, by August 2021, misinformation had resulted in only 50% of the U.S. population being fully vaccinated, posing risks for women of reproductive age as pregnancy can exacerbate the severity of the disease [68]. Despite initial uncertainties, the vaccine received FDA approval for individuals aged 12 and above, inclusive of those pregnant or breastfeeding.

### 4.2. Study Limitations

The study’s limitations include its cross-sectional design, which can depict associations but not causations, potentially leading to challenges in capturing temporal relationships. Participants were sourced primarily from educational databases, possibly overlooking women outside these systems, and the specific focus on those with a history of pregnancy may introduce selection bias. Excluding individuals infected with SARS-CoV-2 may omit crucial insights, while the variability in annual sample sizes and reliance on self-reported data, including an unstandardized survey for pandemic-specific questions, may impact data reliability and comparability. Conducted solely within Romania, the findings’ generalizability to other regions or cultural contexts might be limited. Another limitation of this study includes the utilization of self-developed, unstandardized questionnaires with a 10-point scale, which may harbor low psychometric validity and rely heavily on subjective assessments. While these instruments were deemed necessary for capturing the nuanced perspectives of the studied population regarding contraception during the pandemic, it is acknowledged that responses may not accurately represent objective knowledge or facts. Furthermore, the precision of these measures to reflect the gradations of individual experiences remains uncertain.

## 5. Conclusions

In conclusion, our three-year study on the impact of the COVID-19 pandemic on contraception awareness and mental well-being has revealed several crucial insights. While background characteristics and certain behaviors remained stable, COVID-19 vaccination rates increased significantly over time. Notably, our findings indicate a positive correlation between contraception awareness and mental well-being, with higher awareness associated with improved mental health and reduced anxiety and depression levels during the pandemic. These results underscore the importance of robust contraception education programs in supporting individuals’ overall health, particularly during public health crises. Public health workers can use this information to prioritize comprehensive contraceptive education and support to enhance both physical and mental well-being in times of adversity.

## Figures and Tables

**Table 1:** Comparison of background characteristics of patients surveyed between 2020, 2021, and 2022.

	2020 (*n* = 59)	2021 (*n* = 56)	2022 (*n* = 60)	*p*-Value *
Age, years (mean ± SD) **	20.3 ± 6.6	21.5 ± 6.4	20.8 ± 6.9	0.624
Age range				0.480
15–18	9 (15.3%)	11 (19.6%)	8 (13.3%)	
19–22	17 (28.8%)	20 (35.7%)	15 (25.0%)	
23–25	33 (55.9%)	25 (44.6%)	37 (61.7%)	
Place of residence				0.786
Urban	31 (52.2%)	38 (67.9%)	35 (58.3%)	
Rural	28 (47.8%)	28 (32.1%)	25 (41.7%)	
Education				0.548
High school	17 (28.8%)	20 (35.7%)	24 (40.0%)	
College	17 (28.8%)	19 (33.9%)	15 (25.0%)	
University	25 (42.4%)	17 (30.4%)	21 (35.0%)	
Relationship status				0.442
Single	12 (20.3%)	14 (25.0%)	19 (31.7%)	
In a relationship/married	38 (64.4)	31 (55.4%)	35 (58.3%)	
Prefer not to say	9 (15.3%)	11 (19.6%)	6 (10.0%)	
Substance use				
Currently smoking	13 (22.0%)	19 (33.9%)	22 (36.7%)	0.187
Alcohol use	9 (15.3%)	7 (12.5%)	11 (18.3%)	0.684
Substance use	5 (8.5%)	8 (14.3%)	8 (13.3%)	0.584
COVID-19 vaccinated	-	9 (16.1%)	22 (36.7%)	0.012
Pregnancies				0.852
1	42 (71.2%)	37 (66.1%)	40 (66.7%)	
2	12 (20.3%)	12 (21.4%)	11 (18.3%)	
≥3	5 (8.5%)	7 (12.5%)	9 (15.0%)	
Births				0.632
0	36 (61.0%)	31 (55.4%)	35 (58.3%)	
1	19 (32.2%)	22 (39.3%)	18 (30.0%)	
≥2	4 (6.8%)	3 (5.4%)	7 (11.7%)	
Abortions				0.263
0	24 (40.7%)	20 (35.7%)	29 (48.3%)	
1	22 (37.3%)	29 (51.8%)	24 (40.0%)	
≥2	13 (22.0%)	7 (12.5%)	7 (11.7%)	
Contraceptives used				0.517
None	13 (22.0%)	15 (26.8%)	11 (18.3%)	
Condoms	42 (71.2%)	36 (64.3%)	40 (66.7%)	
Pills	4 (6.8%)	5 (8.9%)	9 (15.0%)	

* Chi-square or Fisher’s exact test; ** ANOVA test; SD—Standard Deviation.

**Table 2:** Unstandardized survey results.

Questions (Answers Given on a Scale from 1 to 10)	2020 (*n* = 59)	2021 (*n* = 56)	2022 (*n* = 60)	*p*-Value *
How confident are you in your current knowledge about contraceptive methods?	6.6 ± 2.4	6.2 ± 3.0	5.9 ± 3.3	0.426
Since the start of the COVID-19 pandemic, did you notice any changes in awareness programs about contraceptive methods?	6.3 ± 3.5	6.6 ± 3.9	7.3 ± 3.1	0.282
To what extent do you believe the pandemic has impacted your access to contraceptive, sexual education, or counseling?	7.1 ± 3.3	7.9 ± 2.6	6.4 ± 3.0	0.029
How frequently have you felt overwhelmed or anxious during the pandemic, excluding academic/work-related issues?	7.4 ± 4.1	7.7 ± 3.2	6.0 ± 3.4	0.025
To what extent have feelings of anxiety or depression deterred you from seeking information or services about contraception during the pandemic?	4.9 ± 4.4	4.6 ± 3.8	5.3 ± 4.0	0.650
How influenced would you rate your overall mental well-being since the beginning of the COVID-19 pandemic?	6.9 ± 4.3	7.6 ± 3.6	5.5 ± 4.1	0.017
How supported do you feel in addressing any challenges or concerns related to contraception during the pandemic?	5.2 ± 4.0	5.5 ± 4.4	4.9 ± 3.8	0.729
To what degree do you believe your mental well-being has influenced your attitude towards contraceptive use and education during the pandemic?	4.7 ± 4.1	5.3 ± 3.8	6.0 ± 4.2	0.217
Considering your knowledge before the pandemic, how well-equipped do you feel now to make informed decisions regarding contraception?	5.4 ± 3.6	5.0 ± 3.2	4.8 ± 4.5	0.685
How significant a role do you believe the pandemic has played in shaping your current perspectives on reproductive health and contraception?	6.1 ± 4.0	6.4 ± 3.5	7.7 ± 3.7	0.049
Do you believe COVID-19 disease can influence your fertility in the future? (yes, %)	38 (64.4%)	29 (51.8%)	21 (35.0%)	0.005 **

* ANOVA test; ** Chi-square or Fisher’s exact test; Scale Explanation.

**Table 3:** SF-36 survey results stratified by COVID-19 pandemic years.

SF-36 (Mean ± SD)	2020 (*n* = 59)	2021 (*n* = 56)	2022 (*n* = 60)	*p*-Value *
Physical	52.1 ± 6.3	55.9 ± 7.0	56.5 ± 6.8	<0.001
Mental	51.4 ± 7.2	52.8 ± 6.8	55.0 ± 6.9	0.019
Total score	53.6 ± 7.9	55.3 ± 7.7	57.5 ± 8.0	0.027

*—ANOVA test; SD—Standard Deviation; SF-36—Short Form Survey (higher scores indicate better health status and quality of life).

**Table 4:** WHOQOL-BREF survey results stratified by COVID-19 pandemic years.

WHOQOL-BREF (Mean ± SD)	2020 (*n* = 59)	2021 (*n* = 56)	2022 (*n* = 60)	*p*-Value *
Physical domain	62.9 ± 16.3	66.0 ± 17.5	68.3 ± 18.0	0.235
Mental domain	60.7 ± 17.0	61.3 ± 16.8	66.5 ± 15.9	0.114
Social domain	53.6 ± 18.2	58.2 ± 18.9	63.0 ± 20.5	0.030
Environmental domain	60.4 ± 15.6	59.3 ± 18.1	64.1 ± 16.9	0.274

*—ANOVA test; SD—Standard Deviation; WHOQOL-BREF—Brief Version of the World Health Organization Quality of Life survey (higher scores indicate better quality of life).

**Table 5:** GAD-7 and PHQ-9 survey results stratified by COVID-19 pandemic years.

Variables (Mean ± SD)	2020 (*n* = 59)	2021 (*n* = 56)	2022 (*n* = 60)	*p*-Value *
GAD-7	7.9 ± 2.6	6.8 ± 3.1	6.5 ± 3.3	0.031
PHQ-9	4.8 ± 2.2	4.3 ± 3.4	3.9 ± 2.8	0.224

*—ANOVA test; SD—Standard Deviation; GAD—General Anxiety Disorder (higher scores indicate higher anxiety symptoms); PHQ—Patient Health Questionnaire (higher scores indicate more severe depression symptoms).

**Table 6:** Correlation analysis between contraception awareness and subjects’ mental well-being.

		Contraception Awareness	Mental Well-Being (SF-36)	Mental Domain (WHOQOL-BREF)	Anxiety Levels (GAD-7)	Depression Levels (PHQ-9)
Contraception Awareness	Rho	1				
	*p*-value	-				
Mental Well-being (SF-36)	Rho	0.324 *	1			
	*p*-value	0.015	-			
Mental Domain (WHOQOL-BREF)	Rho	0.298 *	0.467 **	1		
	*p*-value	0.023	0.001	-		
Anxiety Levels (GAD-7)	Rho	−0.412 **	−0.21	0.558 **	1	
	*p*-value	0.001	0.087	0.001	-	
Depression Levels (PHQ-9)	Rho	−0.389 **	−0.310 **	−0.532 **	0.294 *	1
	*p*-value	0.001	0.001	0.001	0.02	-

*—significant at the 0.05 significance level; **—significant at the 0.01 significance level; Contraception awareness was calculated as a composite score of questions 1–5 from Table 2; SF-36—Short Form (36 questions); WHOQOL-BREF—Brief Version of the World Health Organization Quality of Life survey (higher scores indicate better quality of life); GAD—General Anxiety Disorder (higher scores indicate higher anxiety symptoms); PHQ—Patient Health Questionnaire (higher scores indicate more severe depression symptoms.

## Data Availability

The data presented in this study are available on request from the corresponding author.
